# Impact of Preoperative Gum Chewing on Postoperative Anti-Emetic Use in Robot-Assisted Laparoscopic Surgery for Benign Ovarian Masses: A Prospective, Single-Blinded Randomized Controlled Trial

**DOI:** 10.3390/medicina60071135

**Published:** 2024-07-15

**Authors:** Min Suk Chae, Subin Lee, Youn Jin Choi, Hyun Jung Koh

**Affiliations:** 1 Department of Anesthesiology and Pain Medicine, Seoul St. Mary’s Hospital, College of Medicine, The Catholic University of Korea, Seoul 06591, Republic of Korea; shscms@catholic.ac.kr; 2 Department of Anesthesiology and Pain Medicine, Yeouido St. Mary’s Hospital, College of Medicine, The Catholic University of Korea, Seoul 06591, Republic of Korea; 3 Department of Obstetrics and Gynecology, Seoul St. Mary’s Hospital, College of Medicine, The Catholic University of Korea, Seoul 06591, Republic of Korea

**Keywords:** gum chewing, nausea and vomiting, robot-assisted surgery, female

## Abstract

*Background and Objectives*: Postoperative nausea and vomiting (PONV) is a common issue for females undergoing gynecological surgeries, including those assisted by robotic systems. Despite available prophylactic measures, the incidence of PONV remains high, negatively impacting recovery and increasing healthcare costs. This study evaluates whether preoperative gum chewing reduces the need for anti-emetic drugs in females undergoing robot-assisted laparoscopic surgery for benign ovarian mass. *Materials and Methods*: This prospective, single-blinded, randomized controlled trial enrolled 92 adult females scheduled for robot-assisted laparoscopic surgery to treat benign ovarian mass. Following exclusions, the remaining participants were randomly assigned to either a gum-chewing group or a no-gum-chewing group. The gum-chewing group chewed sugar-free gum for 15 min in the holding area before surgery. The primary outcome measured was the need for anti-emetics to control PONV during the first hour in the post-anesthesia care unit (PACU). Secondary outcomes included the number of anti-emetic requests. No preemptive anti-emetics were administered during surgery. *Results*: Out of the initial 92 patients, 88 were included in the final analysis, with 44 in each group. The incidence of PONV requiring anti-emetics in the PACU was significantly lower in the gum-chewing group (79.5%) compared to the no-gum-chewing group (95.5%). Additionally, the number of anti-emetic requests was higher in the no-gum-chewing group. No postoperative complications such as tooth or jaw pain/injury or gastric content regurgitation were reported. *Conclusions*: Preoperative gum chewing for 15 min immediately before surgery significantly reduced the incidence of PONV in females undergoing robot-assisted laparoscopic surgery for benign ovarian mass. This simple, non-pharmacological intervention improved patient comfort and reduced the need for anti-emetic medications without any adverse effects. Further studies are needed to confirm these findings and to develop guidelines for incorporating preoperative gum chewing into clinical practice.

## 1. Introduction

Postoperative nausea and vomiting (PONV) are frequent complications after surgery, impacting around 30% of surgical patients and over 70% of those with clinical risk factors, including female gender, a history of PONV or motion sickness, non-smoking status, and the use of postoperative opioids [1,2,3,4]. Kawano et al. observed an incidence of approximately 72% for nausea or vomiting in patients undergoing laparoscopic gynecological surgery [5]. Adult patients at risk for PONV include females, those with a history of PONV or motion sickness, non-smokers, and those using postoperative opioids [6,7,8]. Current guidelines recommend multimodal prophylaxis for patients with one or more risk factors [9]. Consensus guidelines suggest the use of multiple antiemetics (such as serotonin 5-HT_3_, dopamine D_2_, histamine H_1_ antagonists, and corticosteroids), propofol-based anesthesia, and non-pharmacologic interventions (including gum chewing) for patients at high risk [10,11,12,13,14].

Abdominal surgery, commonly used to treat various benign and malignant gynecologic conditions, often leads to paralytic ileus. This complication arises from both localized and generalized intestinal inflammation, neurotransmitter release from bowel manipulation, perioperative opioid use, and afferent reflex stimulation due to peritoneal irritation. Common symptoms include nausea, vomiting, abdominal distension, delayed bowel movements, inability to eat, prolonged hospital stays, and increased healthcare costs [15,16,17]. Robot-assisted laparoscopic surgery (RALS), which involves less physical manipulation and smaller incisions than traditional laparotomy, promotes faster recovery of bowel function and reduces the incidence of ileus [18,19,20]. Furthermore, the decreased pain and smaller incisions associated with RALS improve patient mobility, further lowering the risk of ileus. However, the use of CO_2_ for abdominal cavity insufflation can temporarily impair intestinal blood flow and motility, potentially causing ileus. This can also lead to acidosis and electrolyte imbalances, disrupting intestinal smooth muscle function and delaying the return to normal gastrointestinal activity [19,20,21].

Chewing gum, considered a form of sham feeding, can improve intestinal motor and secretory functions. Chewing activates the cephalic–vagal reflex, which enhances intestinal myoelectric activity and triggers the release of substances such as gastrin, neurotensin, pancreatic polypeptides, and duodenal enzymes [22]. The safety and effectiveness of postoperative gum chewing have been confirmed in various abdominal surgeries, including cesarean sections, colectomies, and radical cystectomies [23,24,25]. However, more research is needed to determine the effects of preoperative gum chewing on PONV following RALS for benign ovarian masses [26].

Therefore, we evaluated the evidence supporting the use of preoperative short-term single-cycle gum chewing to reduce anti-emetic requirements and improve PONV management in the post-anesthesia care unit (PACU) following RALS for benign ovarian masses.

## 2. Materials and Methods

### 2.1. Ethical Considerations

This prospective, single-blinded randomized trial received approval on 15 March 2024, from the Institutional Review Board and Ethics Committee of Seoul St. Mary’s Hospital, Catholic University of Korea, a tertiary academic teaching institution (approval number: KC24EISI0138). The study protocol was registered with the Clinical Research Information Service (www.cris.nih.go.kr) under identifier KCT0009517 on 12 June 2024. In alignment with the principles outlined in the Declaration of Helsinki, informed consent was obtained from all participants the day before their surgeries. The research adhered to the Consolidated Standards of Reporting Trials (CONSORT) guidelines, ensuring high ethical and methodological standards.

### 2.2. Study Population

This study included adult female patients undergoing RALS for benign ovarian masses. Inclusion criteria were females aged 19–70 years scheduled for elective RALS for benign ovarian masses and classified as American Society of Anesthesiologists (ASA) Physical Status I or II. Exclusion criteria included patients initially scheduled for robot-assisted surgery but subsequently switched to another surgery type; patients with a history of dental damage, dentures, or loose or crowned teeth; patients with problems chewing, such as temporomandibular joint damage or surgery; patients with a history of head or neck surgery that might cause anatomical or functional issues interfering with gum chewing, such as restricted jaw movement or sensitivity in the surgical area; emergency surgeries; ovarian cancer; and patients who declined to participate in the study.

Of 92 patients, 4 were excluded due to ovarian cancer. Consequently, 88 patients were included in the study and were randomly assigned to one of two groups: 44 patients to the no-gum-chewing (NGC) group and 44 patients to the gum-chewing (GC) group.

### 2.3. Randomization and Blinding

**Figure 1:**
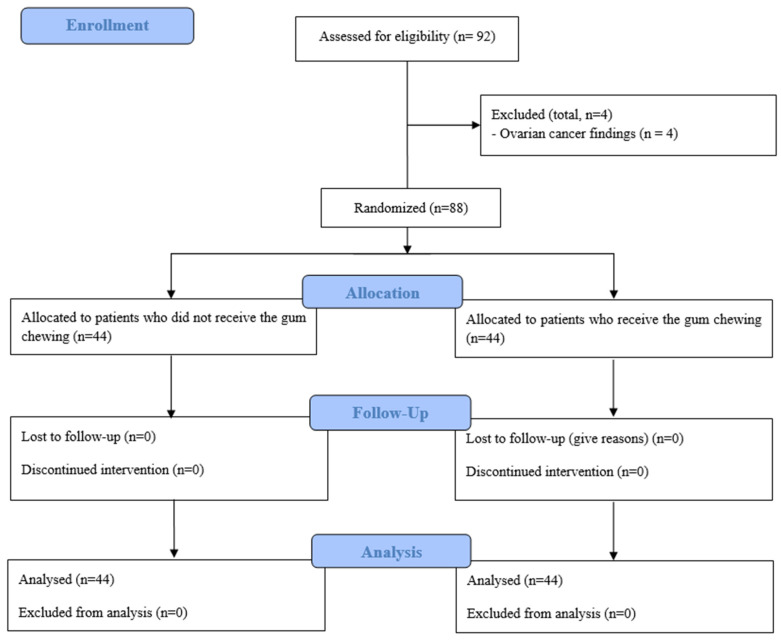
Study flowchart.

Patients were assigned to groups through stratified block randomization, using a web-based generator (www.random.org, accessed on 12 June 2024). The research staff carried out the assignment by opening sequentially numbered, opaque envelopes to determine each patient’s group. This process took place in the surgery waiting room (holding area) and was communicated to the gum preparation room via a sealed envelope. An anesthesia nurse, who was not involved in outcome evaluations, prepared the sugar-free gum according to the assigned numbers. To maintain impartiality, all anesthesiologists and healthcare providers responsible for assessing postoperative outcomes remained blinded to the group assignments (Figure 1).

### 2.4. Gum Chewing in the Holding Area

Patients in the GC group were instructed to chew the gum for 15 min while in the holding area prior to entering the operating room. The gum used in the study was sugar-free mint, chosen for its potential benefits in reducing nausea and vomiting [27]. After this period, they handed the gum to the anesthesia nurse to confirm compliance. Although the patients were aware of their group assignments, medical staff in the PACU remained blinded, ensuring that healthcare providers were unaware of group allocations.

### 2.5. PONV Measurement in the PACU

The primary outcome measured was the need for anti-emetics due to nausea and vomiting during the first hour in the PACU, with the frequency of these requests designated as the secondary outcome. No preemptive anti-emetics were administered during surgery. If needed in the PACU, nurses first administered palonosetron hydrochloride 0.075 mg (Aloxi; CJ Healthcare, Seoul, Republic of Korea), followed by ondansetron 4 mg (Zofran; Hana Pharm, Seoul, Republic of Korea) if necessary. Palonosetron was used as the first anti-emetic drug infusion in response to PONV during the first hour in the PACU. If the initial palonosetron infusion did not adequately control the PONV, ondansetron was administered as the second anti-emetic drug infusion. The sequential use of these anti-emetic drugs reflects the severity of PONV.

### 2.6. Robot-Assisted Surgery and General Anesthesia

Both robot-assisted ovary cystectomy and robot-assisted salpingo-oophorectomy involved positioning patients in the lithotomy position under general anesthesia. The surgical field was prepared, and pneumoperitoneum was established with CO_2_ via a trocar inserted near the umbilicus. Additional trocars were placed under direct vision for robotic instrument access. The da Vinci Surgical System (Intuitive Surgical Inc.; Sunnyvale, CA, USA) was docked, and the surgeon operated the robotic arms from a console. In ovary cystectomy, the ovarian cyst was meticulously dissected and removed, preserving normal ovarian tissue. In salpingo-oophorectomy, the ovary and fallopian tube were detached after transecting the infundibulopelvic and ovarian ligaments. Hemostasis was achieved using bipolar cautery, and the excised tissues were retrieved through one of the port sites. Procedures concluded with the release of pneumoperitoneum and closure of the incisions in layers.

Balanced general anesthesia was induced with propofol (2 mg/kg) and rocuronium (0.8–1 mg/kg), followed by tracheal intubation. Anesthesia maintenance involved 2% propofol and 2 mg remifentanil, using effect-site control and Minto’s model to achieve a bispectral index (BIS) of 40–60 and to keep systolic blood pressure within 20% of the baseline value. Monitoring during the procedure included electrocardiography, pulse oximetry, noninvasive blood pressure measurement, and BIS monitoring. Upon surgery completion, sugammadex (4 mg/kg) was administered to reverse the neuromuscular blockade under 100% oxygen.

Postoperative analgesia was managed with 50 µg intravenous fentanyl for peak visual analog scale (VAS) scores > 6, as assessed by anesthesiologists in the PACU and physicians in the ward, none of whom were involved in the trial. Nursing staff documented all analgesic medications administered.

### 2.7. Clinical Variables

Preoperative data collected included age, type of operation (robot-assisted laparoscopic [RAL] ovary cystectomy or RAL salpingo-oophorectomy), body mass index (BMI), ASA physical class, history of motion sickness or PONV, current smoking status, diabetes mellitus (DM), and hypertension (HBP). Patients with a history of motion sickness or PONV were specifically included to assess the efficacy of preoperative gum chewing in a high-risk population. Intraoperative data included the duration of surgery, total remifentanil infusion, total fluid infusion, and total hemorrhage. Postoperative pain-related outcomes in the PACU included rescue fentanyl infusion and peak VAS scores. Complications related to gum chewing, such as tooth and jaw pain or injury and regurgitation of gastric contents in the oral cavity, were monitored.

### 2.8. Sample Size and Statistical Analysis

The study’s sample size was initially calculated using data from 20 patients who underwent RALS for benign ovarian masses. In this preliminary study, the incidence of PONV requiring anti-emetics in the PACU was 70% (7 out of 10 patients) in the NGC group and 40% (4 out of 10 patients) in the GC group. With a 1:1 allocation ratio, each group needed ≥42 participants to achieve 80% statistical power while maintaining a 5% type I error rate. To compensate for an expected dropout rate of ~10%, the study aimed to enroll a total of 92 participants to ensure robustness and reliability in the findings.

The normality of data distribution was evaluated using the Shapiro–Wilk test. For data that followed a normal distribution, unpaired *t*-tests were utilized, while the Mann–Whitney U-test was applied to data that did not follow a normal distribution. Categorical data were analyzed with Pearson’s *χ*^2^ test or Fisher’s exact test, as appropriate. Results are expressed as means ± standard deviations or as numbers with percentages, where applicable. A *p*-value of less than 0.05 was considered statistically significant. All statistical analyses were conducted using SPSS for Windows (version 24.0; IBM, Armonk, NY, USA).

## 3. Results

The final study cohort included 88 female participants with an average age of 31.3 ± 7.4 years and a BMI of 22.1 ± 2.3 kg/m^2^. All surgeries addressed benign ovarian masses. The most frequent diagnosis was an endometrial cyst (n = 36, 40.9%), followed by a dermoid cyst (n = 24, 27.3%) and benign teratoma (n = 11, 12.5%). Among the participants, 63 (71.6%) underwent RAL ovary cystectomy, and 25 (28.4%) underwent RAL salpingo-oophorectomy. Regarding ASA physical status, 48 participants (54.5%) were classified as ASA I and 40 (45.5%) as ASA II. The prevalence of DM was 6.8% (n = 6) and that of HBP was 2.3% (n = 2). Motion sickness was reported by 26 participants (29.5%), and 13 (14.8%) were current smokers.

**Figure 2:**
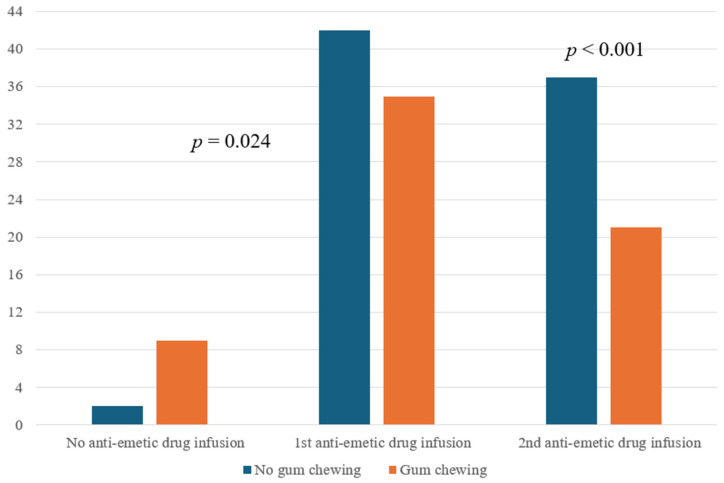
Anti-emetic drug needs of patients in the PACU comparing the preoperative gum-chewing and no gum-chewing groups.

Preoperative and intraoperative findings, as well as postoperative pain-related outcomes (including rescue fentanyl infusion and peak VAS scores), were similar between the groups (Table 1). In the PACU, the need for anti-emetics due to PONV was higher in the NGC group than in the GC group. In addition, the frequency of anti-emetic requirements was greater in the NGC group (Table 2 and Figure 2). There were no postoperative complications such as tooth or jaw pain/injury or regurgitation of gastric contents, confirmed by direct observation immediately after intubation and extubation by attending anesthesiologists not involved in the study.

## 4. Discussion

The primary findings of our study indicate that chewing gum for 15 min immediately before surgery can help reduce nausea and vomiting in female patients with benign ovarian masses undergoing minimally invasive RALS. This practice may decrease the need for anti-emetic drugs without leading to complications such as jaw pain, tooth damage, or postoperative gastric regurgitation.

Research indicates that multiple cycles of gum chewing after surgery can help restore bowel function and decrease the incidence of PONV. A recent systematic review and meta-analysis of nine randomized controlled trials [28] demonstrated that gum chewing significantly accelerates the recovery of gastrointestinal function in women undergoing laparoscopic gynecologic surgery. However, this review did not find significant benefits of gum chewing concerning the time to first postoperative mobilization, the occurrence of postoperative ileus, or the length of hospital stay. A broader review of several randomized controlled trials assessed the effect of gum chewing on postoperative gastrointestinal recovery across various surgeries, including gynecologic surgery, cesarean sections, hepatic surgery, vascular surgery, and urologic surgery. The findings indicated that gum chewing effectively sped up gastrointestinal recovery, particularly in non-gastrointestinal surgeries. Despite this, the advantages of gum chewing after gastrointestinal surgery are still debated [29]. A Cochrane systematic review underscored the potential benefits of postoperative gum chewing in enhancing gastrointestinal recovery. However, the studies included in this review, which mainly focused on colorectal surgery and cesarean sections, were often small and of low quality, revealing methodological limitations [30].

It is important to recognize that many elements of the Enhanced Recovery After Surgery (ERAS) protocol also target postoperative ileus, potentially reducing the extra benefits of gum chewing when used alongside ERAS protocols [30]. Another meta-analysis, which reviewed eight randomized controlled trials conducted between 2013 and 2017 with 1077 women, found that gum chewing significantly shortened the time to first flatus and defecation following gynecologic surgery and reduced hospital stays [31]. Additionally, a systematic review of six randomized controlled trials with 669 participants evaluated the impact of gum chewing on postoperative ileus after gynecologic cancer surgery. The findings indicated that postoperative gum chewing facilitated faster gastrointestinal recovery and decreased the incidence of postoperative ileus. It was also linked to a quicker onset of first flatus and bowel movement, as well as shorter, complication-free hospital stays [32].

While systematic reviews indicate the potential benefits of gum chewing in promoting postoperative gastrointestinal recovery, its specific impact on RAL gynecologic surgery remains uncertain. The less invasive nature of RAL procedures may lead to reduced gastrointestinal disruption compared to more invasive surgeries, which could affect the effectiveness of gum chewing in these cases [19,20,33]. Despite the advancements in minimally invasive robotic surgeries, the management of PONV remains crucial for patient comfort and recovery. The incidence of PONV in laparoscopic procedures, including robotic surgeries, remains high due to factors such as CO_2_ insufflation and patient positioning [19,20,21,25,33]. Therefore, effective strategies to manage PONV are still needed to enhance patient outcomes and satisfaction. Despite the use of antiemetic prophylaxis such as corticosteroids (e.g., dexamethasone), 5HT_3_ serotonin receptor antagonists, and D_2_ dopamine receptor antagonists, the incidence of PONV after robot-assisted mitral valve replacement surgery remains high. Most PONV episodes occur within the first 12 h postoperatively, with 88% of episodes happening within 24 h, indicating that scheduled postoperative treatment might be more effective than rescue treatment [19]. The exact cause of the high PONV rate following such surgery is unclear, but it may be related to excessive stimulation of vagal receptors in the surgical field. PONV rates are higher in laparoscopic procedures, likely due to irritation from capnothorax [34]. PONV is also common in patients undergoing robotic hysterectomy, despite the use of various antiemetics. Maintaining muscular tissue oxygen saturation (SmtO_2_) above 70% and higher than baseline levels during robotic hysterectomy may serve as a therapeutic prophylaxis for PONV [20]. In RAL radical prostatectomy, propofol-based anesthesia may be more effective than inhalation-based anesthesia for preventing PONV during the early postoperative period, regardless of patient-related risk factors [33]. Despite the minimally invasive nature of robot-assisted surgery, PONV remains a significant concern. A multimodal antiemetic strategy, including the use of antiemetic drugs, monitoring of muscular saturation, and specific anesthesia techniques, is essential. Unlike previous studies, our research highlights the benefits of a simple and easily implementable preoperative intervention, gum-chewing, as a non-pharmacological and cost-effective method to reduce PONV.

The topic of allowing gum chewing during the preoperative fasting period remains debated. Research has shown that gum chewing does not significantly increase gastric volume when comparing preoperative and postoperative groups. A meta-analysis of four studies did note a small but statistically significant increase in gastric fluid volume, though this increase is likely clinically insignificant in terms of aspiration risk. Additionally, gum chewing does not affect gastric acidity, with pH levels remaining unchanged. Importantly, none of the studies reported instances of regurgitation or aspiration of gastric contents during surgery [35]. Studies also suggest that chewing gum before surgery may accelerate postoperative gastrointestinal recovery [36]. It activates chemical and mechanical receptors, promotes the secretion of digestive juices, enhances gastrointestinal activity, and stimulates the release of gastrin and other gastrointestinal hormones, similar to the effects seen with postoperative gum chewing [30]. Furthermore, vagal nerve stimulation, increased acetylcholine release, splanchnic vascular dilation, improved blood supply, and increased gastric peristalsis can alleviate symptoms such as bloating and constipation, aiding postoperative gastrointestinal recovery and reducing discomfort and pain associated with delayed recovery. These effects contribute to overall improved postoperative outcomes [22,37]. Thus, preoperative gum chewing likely does not increase the risk of pulmonary aspiration of gastric contents and could be a safe practice to reduce PONV and the need for antiemetic medication in female patients undergoing RAL surgery for benign ovarian masses. Furthermore, the cost-effectiveness of preoperative gum chewing is advantageous, considering it is a low-cost intervention that can potentially reduce the need for additional antiemetic medications. Given the high costs associated with antiemetic drugs and the potential adverse effects of their use, incorporating gum chewing as a non-pharmacological intervention could provide significant economic benefits and improve patient care.

We investigated the effects of preoperative gum chewing to evaluate its potential as a simple, non-pharmacological intervention for reducing PONV in patients undergoing RALS for benign ovarian masses. Our study specifically focused on preoperative gum chewing because it activates the cephalic–vagal reflex, enhancing intestinal motility and secretion, which may mitigate the onset of PONV immediately after surgery. Previous studies have demonstrated the benefits of postoperative gum chewing in various surgeries, but preoperative gum chewing remains underexplored. Studies have shown that chewing gum postoperatively can reduce ileus and improve gastrointestinal recovery in abdominal surgeries [22,27,28,37]. By investigating preoperative gum chewing, we aimed to determine if similar benefits could be observed earlier in the perioperative period, potentially reducing the immediate need for anti-emetic medications. Our results indicate that preoperative gum chewing significantly reduces the incidence of PONV in the first hour post-surgery, thereby decreasing the reliance on anti-emetic drugs. This finding aligns with the cephalic–vagal reflex theory, which suggests that chewing stimulates gastric and pancreatic secretions, aiding in gastrointestinal motility [12,16,17,18,34]. Patients with a history of motion sickness or PONV were included in the study as they represent a high-risk group for PONV. The inclusion of these patients allows for a comprehensive assessment of the efficacy of preoperative gum chewing in reducing PONV across a broad spectrum of patients. This factor could potentially affect the results, and further studies may be needed to isolate the effects of gum chewing in different risk groups.

Future research should compare the effects of preoperative, postoperative, and combined preoperative plus postoperative gum chewing to determine the most effective timing and duration for reducing PONV. The rationale behind this combined approach is that while preoperative gum chewing activates the cephalic–vagal reflex, enhancing intestinal motility and secretion, postoperative gum chewing could further promote gastrointestinal recovery and reduce ileus. Previous research has shown that postoperative gum chewing accelerates the recovery of gastrointestinal function in various surgeries, including gynecologic and colorectal surgeries [27,28,31,36]. By combining preoperative and postoperative gum chewing, we hypothesize that there could be a synergistic effect, resulting in a more significant reduction in PONV and improved overall gastrointestinal recovery. Such studies could provide a comprehensive understanding of the role of gum chewing in PONV management and establish standardized protocols for its implementation.

The European Society of Anesthesiology advises that operations should not be canceled or delayed if patients are chewing gum, sucking a boiled sweet, or smoking immediately before anesthesia induction [38]. However, removing chewing gum before anesthesia induction is practical to prevent airway obstruction, so it is recommended to include a specific question about gum chewing on preoperative checklists [39]. If patients chew gum during the pre-anesthetic fasting period, there should be a step to ensure the gum is removed before entering the operating room. In our study protocol, a nurse in the holding area confirmed gum removal before the patient was taken to the operating room, ensuring no airway issues during the study period.

Our study has several limitations. First, because preoperative gum chewing and non-gum-chewing are easily distinguishable, ensuring blinding was challenging. Second, the small sample size in our trials may have affected the internal validity and obscured the true effects of, the outcomes. Future studies should aim to include a larger sample size to enhance the reliability and validity of the findings. Increasing the number of participants would help to confirm the results observed in our study and provide more robust evidence for the efficacy of preoperative gum chewing in reducing PONV. A larger sample size would also allow for more detailed subgroup analyses, potentially uncovering differences in efficacy among various patient demographics and surgical contexts. Third, we only measured PONV requiring antiemetics, potentially overlooking mild PONV cases that did not require such interventions. Future studies should include more detailed assessments of PONV severity and duration, not just the requirement for anti-emetic drugs. Utilizing a standardized scoring system, such as the Baxter Retching Faces (BARF) scale or the Visual Analog Scale (VAS) for nausea, could provide a more comprehensive evaluation of PONV. These tools would allow for consistent and quantifiable measurements of nausea intensity and duration, thereby enhancing the robustness of the data and enabling better comparisons across studies. Including these detailed assessments would provide a clearer understanding of the efficacy of preoperative gum chewing in managing PONV and its impact on patient recovery. Fourth, we conducted the preoperative gum-chewing intervention in the holding area just before surgery, limiting the duration to a single 15 min cycle. Fifth, our study is the absence of an additional control group receiving a different non-pharmacological intervention. Future studies should consider incorporating multiple control groups to compare the efficacy of various non-pharmacological interventions for PONV management. Finally, due to the complexity and invasiveness of ovarian cancer surgery, our study only included robot-assisted surgery for benign ovarian masses. Future research should explore the effects of multiple and longer durations of preoperative gum chewing during the preoperative period.

## 5. Conclusions

Chewing gum for 15 min immediately before surgery appears to be a safe and effective method to reduce PONV in female patients undergoing minimally invasive robot-assisted surgery for benign ovarian masses. This brief preoperative gum chewing can reduce PONV and the reliance on related medications, thereby enhancing patient comfort and improving the efficiency and quality of care provided by PACU staff. Specifically, for female patients undergoing RAL procedures, reducing PONV can also offer cost benefits. While our study highlights potential advantages, further large-scale, blinded, and standardized trials are necessary to validate these findings and establish consistent protocols for preoperative gum chewing in clinical practice.

## Figures and Tables

**Table 1:** Demographic comparison of the preoperative gum-chewing and no-gum-chewing groups.

Group	No Gum Chewing	Gum Chewing	*p* Value
n	44	44	
***Preoperative findings***			
Age (years)	31 ± 7	32 ± 8	0.587
Operation type			
Robot-assisted laparoscopic ovary cystectomy	34 (77.3%)	29 (65.9%)	0.237
Robot-assisted laparoscopic salpingo-oophorectomy	10 (22.7%)	15 (34.1%)	
Etiology			
Teratoma	5 (11.4%)	6 (13.6%)	0.723
Serious cystadenoma	1 (2.3%)	1 (2.3%)	
Polycystic ovary syndrome	1 (2.3%)	0 (0.0%)	
Mucinous cystadenoma	4 (9.1%)	6 (13.6%)	
Follicular cyst	1 (2.3%)	3 (6.8%)	
Endometrial cyst	21 (47.7%)	15 (34.1%)	
Dermoid cyst	11 (25.0%)	13 (29.5%)	
Ovary operation site			
Left	16 (36.4%)	23 (52.3%)	0.2
Right	12 (27.3%)	12 (27.3%)	
Both	16 (36.4%)	9 (20.5%)	
Body mass index (kg/m^2^)	21.7 ± 2.2	22.5 ± 2.5	0.101
ASA physical class			
Class I	21 (47.7%)	27 (61.4)	0.199
Class II	23 (52.3%)	17 (38.6%)	
History of motion sickness/PONV	11 (25.0%)	15 (34.1%)	0.35
Current smoker	6 (13.6%)	7 (15.9%)	0.764
Diabetes mellitus	4 (9.1%)	2 (4.5%)	0.676
Hypertension	1 (2.3%)	1 (2.3%)	>0.999
***Intraoperative findings***			
Surgery duration (min)	143.0 ± 49.0	159.1 ± 50.4	0.133
Total remifentanil infusion (mg)	0.6 ± 0.3	0.5 ± 0.4	0.87
Total fluid infusion (mL)	411.8 ± 167.2	387.6 ± 197.6	0.537
Total hemorrhage (mL)	101.4 ± 55.5	107.5 ± 61.2	0.625
***Pain findings in the PACU***			
Rescue fentanyl infusion (mcg)	59.1 ± 19.5	53.4 ± 12.7	0.109
Peak VAS	4.5 ± 0.8	4.2 ± 0.7	0.12

**Abbreviations:** ASA, American Society of Anesthesiologists; PONV, postoperative nausea and vomiting; VAS, visual analog scale; PACU, post-anesthesia care unit. Values are expressed as mean ± SD and number (proportion).

**Table 2:** Comparisons of PONV severity and anti-emetic drug requirements in the PACU for the preoperative gum-chewing and no-gum-chewing groups.

Group	No Gum Chewing	Gum Chewing	*p* Value
n	44	44	
PONV severity			
*Mild to moderate PONV*			
No anti-emetic drug infusion	2 (4.5%)	9 (20.5%)	0.024
1st anti-emetic drug infusion (palonosetron only)	42 (95.5%)	35 (79.5%)	
*Severe PONV*			
2nd anti-emetic drug infusion (ondansetron needed)	37 (84.1%)	21 (47.7%)	<0.001

**Abbreviation:** PONV, postoperative nausea and vomiting, PACU, post-anesthesia care unit. Values are expressed as numbers (proportion).

## Data Availability

Data are contained within the article.

## Abbreviations

PONV: postoperative nausea and vomiting; PACU, post-anesthesia care unit; RALS, robot-assisted laparoscopic surgery; ASA, American Society of Anesthesiologists; TMJ, temporomandibular joint; VAS, visual analog scale; DM, diabetes mellitus; HBP, hypertension; BMI, body mass index.
